# A Comparison of two Maps of the Human Neocortex: the multimodal MRI-based parcellation of Glasser et al. (2016a), and the myeloarchitectonic parcellation of Nieuwenhuys and Broere (2023), as a first step toward a unified, canonical map

**DOI:** 10.1007/s00429-024-02860-x

**Published:** 2024-11-22

**Authors:** Rudolf Nieuwenhuys, Matthew F. Glasser

**Affiliations:** 1 The Netherlands Institute for Neuroscience, Royal Netherlands Academy of Arts and Sciences, Meibergdreef 47, 1105 BA Amsterdam, The Netherlands; 2 Department of Medical Imaging, Anatomy, Radboud University Medical Center, P.O. Box 9101, 6500 HB Nijmegen, The Netherlands; 3 Departments of Radiology, Neuroscience, and Biomedical Engineering, Washington University Medical School, St. Louis, MO USA

**Keywords:** Architectonics, Canonical map, Cerebral cortex, Cortical areas, Cytoarchitectonics, Myeloarchitectonics, Neuroimaging

## Abstract

The first, introductory part of this paper presents an overview of the long quest for a universal map of the human cortex, useful as a standard reference for all remaining studies on this brain part. It is pointed out that such a map does still not exist, but that systematic comparison of some recently produced 3D maps may well be conducive toward this important goal. Hence, the second part of this article is devoted to a detailed comparison of two of such maps, the multimodal MRI-based parcellation of Glasser et al. (Nature 536:171–178, 2016) and the myeloarchitectonic parcellation presented by Nieuwenhuys and Broere (Brain Struct Funct 228:1549–1559, 2023), with the specific aim to detect areal concordances between these two maps. In the search for these concordances, the following three criteria were used: (1) the relative or topological position of the various areas, (2) the relation of the areas to particular invariant sulci, and (3) the overall myelin content of the areas. In total 61 concordances were detected, most of which were located in the frontal and parietal lobes. These concordances were recorded in standard views of the two maps compared (Figs. 5, 6, 7, 8), as well as in Table 1. We consider these findings as a first step towards the creation of a unified, consensus (canonical) parcellation of the human neocortex.

## Introduction

By the end of the nineteenth century it was known that the cerebral cortex is not homogeneous, but is rather composed of fields or areas of different structure (Meynert 1868; Kaes 1893, Hammarberg 1895). Moreover, there was clinical and experimental evidence, indicating that the cortex harbours centres with clearly different functions (Broca 1865; Wernicke 1874; Henschen 1894). During the twentieth century, numerous architectonic maps of the human cerebral cortex were published (e.g. Brodmann 1909; Von Economo and Koskinas 1925, Baily and Von Bonin 1951), but these maps showed considerable differences, and the ultimate goal, the production of a unified, canonical map of the human cortex, was by no means achieved.

An important new impetus to human brain mapping has been the advent of brain imaging techniques, such as positron emission tomography (PET) and functional magnetic resonance imaging (fMRI). These techniques render it possible to spot loci of activation non-invasively in the living brain. It stands to reason that the results of such studies can only be qualified as scientific, if the cortical areas, responsible for these activities, are determined. Initially, such determinations were provisionally performed by transferring the activated loci to a three-dimensional version of Brodmann’s map, produced by Talairach and Tournoux (1988, 1993). However, it soon became clear that this map does not provide the neuromorphological precision and accuracy for an adequate mapping of fMRI data (Geyer et al. 2011; Glasser et al. 2016b). It may be added in this context that, in the mean time, it had become increasingly clear that the Vogt-Vogt concept, according to which the human cortex is composed of about 200 discrete, juxtaposed structural and functional modules or units, is essentially correct (Vogt and Vogt 1919, Van Essen et al. 2012 and Nieuwenhuys 2013).

In what follows, we will briefly indicate some results of the current, largely MRI-driven or MRI-inspired, mapping studies on the human cerebral cortex.

1. Karl Zilles (†2021), Katrin Amunts, and their numerous associates started in the mid 1990s a new research program, aimed at producing a probabilistic map of the human cerebral cortex, based on quantitative cytoarchitectonic and receptorarchitectonic analyses of the brains of ten individuals (Amunts and Zilles 2015; Amunts et al. 2007, 2020; Palomero-Gallagher and Zilles 2018; Zilles and Amunts 2009). At present, about 80% of the cortical surface has been covered within the frame of this program. Some of these maps were mapped onto cortical surface meshes and aligned across individuals on the cortical surface using cortical folding patterns (Fischl et al. 2008), allowing precise surface mesh-based comparisons to MRI-based measures.
2. Improvements in the quality of in vivo MRI scanning rendered it possible to visualize structural features of the human cortex. Particularly myelinated fibres appeared to give excellent MRI contrast (Glasser and Van Essen 2011; Geyer et al. 2011). In order to obtain histology-based data on the myeloarchitecture of the human cortex, that could be compared with the structural MRI findings just mentioned, one of us scrutinized the detailed myeloarchitectonic publications of the Vogt-Vogt school (Nieuwenhuys 2013) and succeeded in creating complete 2D and 3D myeloarchitectonic maps of the human neocortex (Nieuwenhuys et al. 2015a,b; Nieuwenhuys and Broere 2023), as well as a map showing the estimated overall myelin content of the individual architectonic areas (Nieuwenhuys and Broere 2017).
3. Finally, advances in accurately mapping high resolution brain functional MRI to cortical surfaces and in accurate cross-subject functional alignment enabled Glasser et al. (2016a) to publish a multimodal surface-mesh based 3D parcellation of the human cortex, based on structural- and functional MRI data derived from the Human Connectome Project (HCP).

We are convinced that progress in the direction of the creation of the *universal, canonical map of the human cerebral cortex* mentioned before, requires a detailed, systematic comparison of the maps just discussed. Hence, the next part of this paper is devoted to a comparison of two of these maps.

## Material

The maps to be compared are the 3D multimodal, MRI-based map, produced by Glasser et al. (2016a) and the 3D myeloarchitectonic map of Nieuwenhuys and Broere (2023), derived from data from the Vogt-Vogt school. The Glasser et al. map is, as already mentioned, based on multimodal MRI images from the Human Connectome Project.

The authors delineated 180 areas per hemisphere, bounded by sharp changes in cortical thickness, relative myelin content, connectivity and functional specificity, in a group average of 210 healthy young adults. Glasser et al. (2016a) also compared, in a preliminary way, the results of their parcellation, with those of numerous previous authors. They found that 83 of their 180 areas had been previously described and that hence 97 areas were new.

The nomenclature employed by Glasser et al. is complex and was chosen to preserve correspondences with prior literature. Some areas are described by numerals (*e.g*. areas 1, 2, 4). These numbers are derived from Brodmann’s (1909, 1914) numbering of cytoarchitectonic areas, though this is not always stated explicitly. Other areas are indicated by all-caps letters (*e.g.* MST, standing for Medial Superior Temporal Area, or FFC, standing for Fusiform Face Complex). Still other areas are indicated by combinations of letters and numbers (*e.g.* V2, standing for Second Visual Area, or TPOJ3, standing for TemporoParietoOccipital Junction 3). Lower case letters typically represent subdivisions of a once-larger parcel, and they signify topographical positions within the brain (d for dorsal, r for rostral etc.). Finally, numerals are used for fine-grained parcellations that cannot be readily characterized by the topographical parcellations indicated above (*e.g.* POS1, TPOJ3). Glasser et al. identify their parcellation as parcellation version 1.0, because they anticipate future refinements as better data become available.

The map of Nieuwenhuys and Broere (2023) is a myeloarchitectonic map, based on four publications from the Vogt-Vogt school, each dealing with one of the telencephalic lobes: Strasburger (1937; frontal lobe), Batsch (1956; parietal lobe), Lungwitz (1937; occipital lobe) and Hopf (1954; temporal lobe). The authors delineated, just like Glasser et al. (2016a), 180 areas per hemisphere. Unfortunately, recently doubt has arisen among us, with regard to the correctness of the parcellation of the lateral and superior views of the frontal lobe in our original 3D map (Nieuwenhuys and Broere 2023: Figs. 4A, B). A preliminary reanalysis of our material showed that the various areas situated in the precentral region of these two views are much too wide (see the section Results for details). Because of these discrepancies, we decided to replace the lateral and superior views of the frontal lobe in our original 3D map, provisionally by 2D views, showing the results of our reanalysis (Figs. 5A, B).

**Fig. 1:**
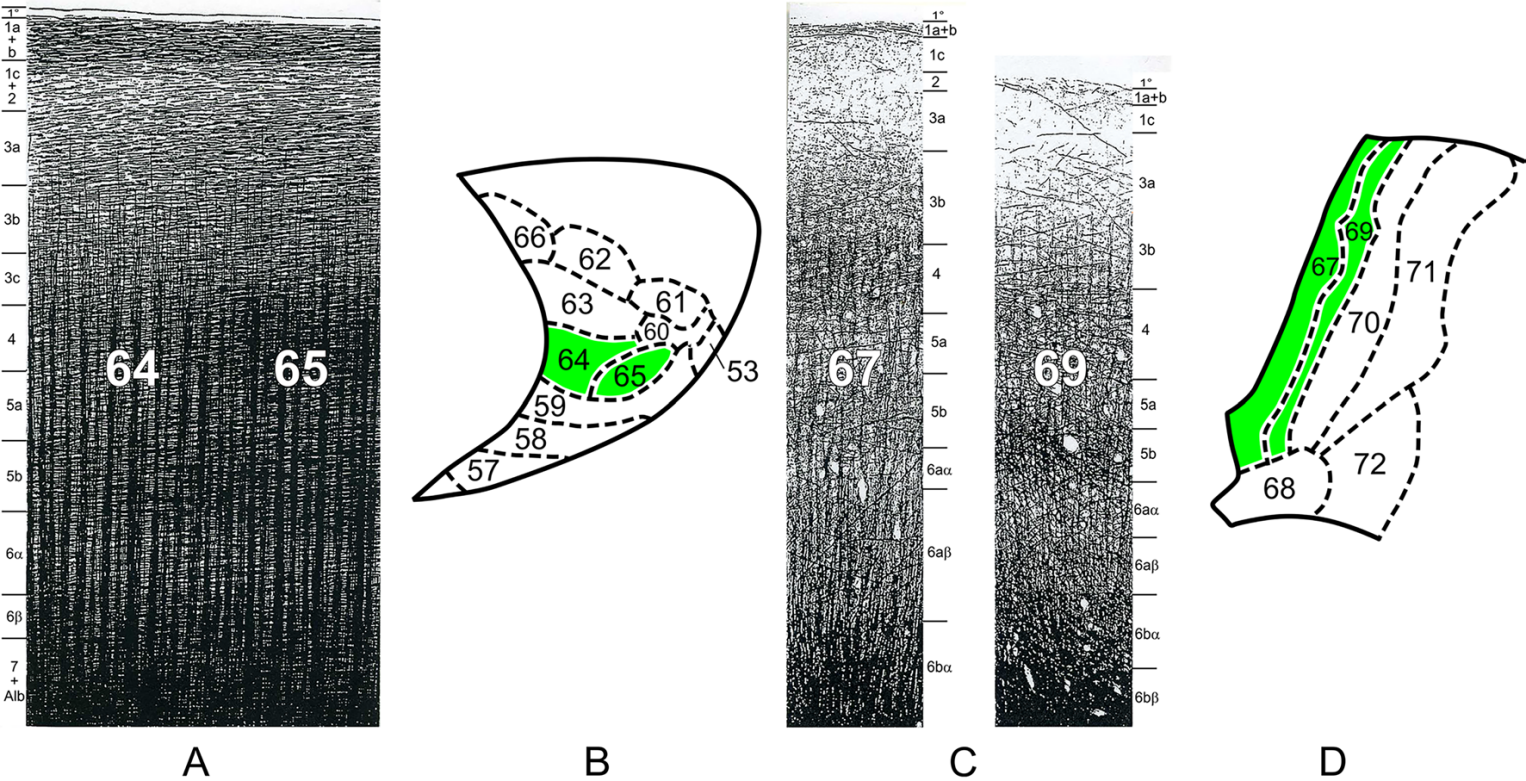
Drawings based on Weigert preparations, showing the myeloarchitecture of two sets of adjacent areas, viz*.* the orbitofrontal areas 64 and 65, derived from Strasburger (1937) (**A**), and the postcentral areas 67 and 69, derived from Vogt (1911) (**C**). The schematics **B** and **D** show the position of these areas

Just for orientation, myeloarchitectonics is a morphological discipline, aiming to subdivide the cerebral cortex on the basis of local differences in the size, orientation and arrangement of the myelinated fibres in that brain part. To give an impression of such differences, we present in Fig. 1 two sets of two adjacent myeloarchitectonic fields.

As regards nomenclature, the designation of the various myeloarchitectonic areas with simple Arabic numbers, introduced by Vogt (1910, 1911) for the frontal and parietal cortices, has been extended here over the entire neocortex.

## Method

We are well aware that, currently, the method of choice for the comparison of two different maps of the cortex includes (1) the transformation of the two maps towards a common template; (2) the preparation of mesh-files of the two transformed maps, and (3) comparison of the two mesh-files by direct superposition. However, unfortunately, we failed to obtain a useful mesh-file of the Nieuwenhuys-Broere (NB) map. Consequently, we had to fall back to the classical mode of comparison: two experienced neuroanatomists, sitting side by side, carefully inspecting the two maps to be compared, in search for concordances. In the search for such concordances between areas or sets of areas in the two maps (see below), the following three criteria were used: The relative or topological position of the various areas, (2) the relation of the areas to particular ‘invariant’ cerebral sulci, and (3) the relative myelin content of the various areas. The relative or topological position represents our principal criterion. It played a dominant role in all of our decisions. The relation to invariant sulci and the relative myelin content represent auxiliary criteria, playing a role in only a limited number of cases.

**Fig. 2:**
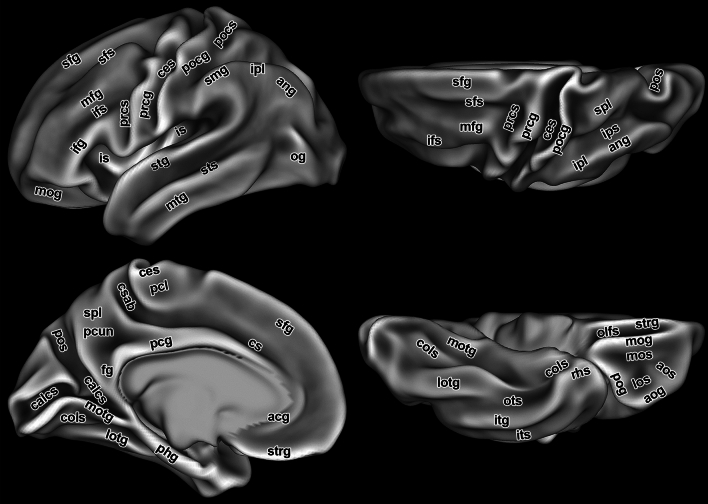
Group average midthickness surface of 1071 multimodally aligned young adults from the Human Connectome Project with an average surface curvature map overlayed. This figure shows the invariant sulci after cortical areas are precisely aligned (*i.e*., those sulci that correlate with cortical areas – the other sulci are blurred away). Some locations such as the central sulcus and the calcarine sulcus have very sharp folding patterns, but others, such as the prefrontal cortex or inferior lateral parietal cortex, have the folds blurred away due to extremely high individual variability

**Fig. 3:**
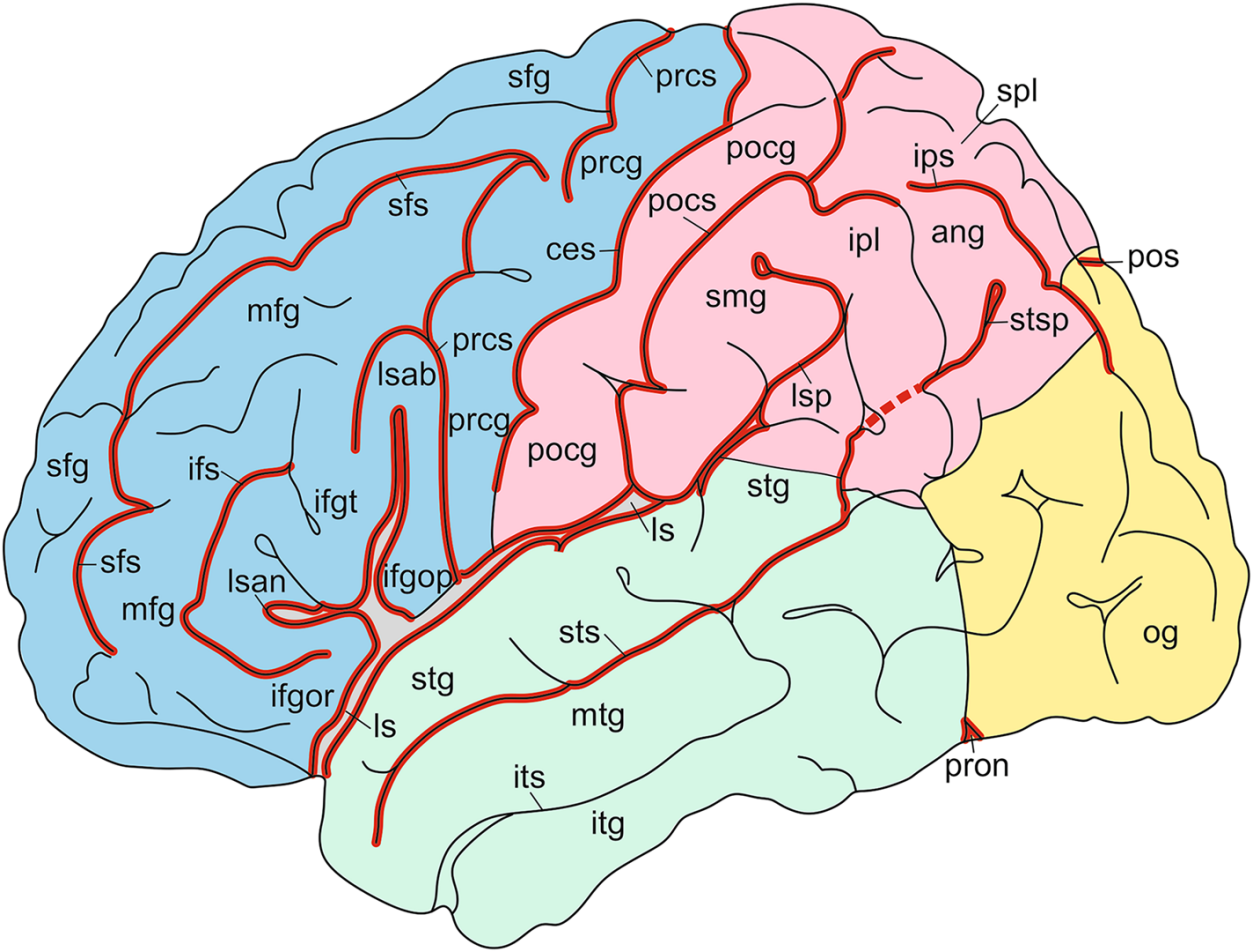
Lateral view of the Colin brain, showing the ‘invariant’ sulci in red. This figure is derived from the atlas of the Colin brain, published by Nieuwenhuys et al. (2015a, b)

**Fig. 4:**
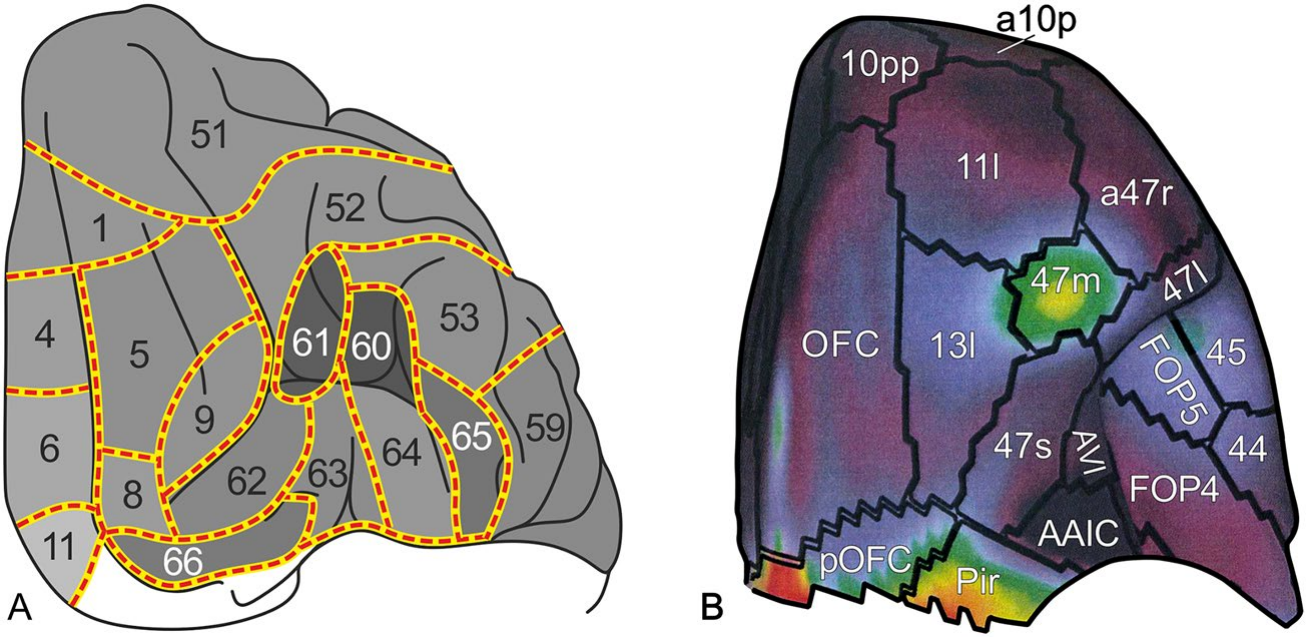
A heavily myelinated locus in the orbitofrontal cortex. In the NB map, this spot comprises two areas: 60 and 61 (**A**). In the HCP map, it consists of a single area: 47m (**B**). Fig. **A** is derived from Nieuwenhuys and Broere (2017). In this Fig. the overall density of the myelin in the individual areas is indicated according to a scale ranging from black to light grey. Fig. **B** is adapted from the Fig. 23 of the Supplementary Neuroanatomical Results from Glasser et al. (2016a). Here, the scale indicating the overall myelin density ranges from red (densely myelinated), via yellow and green to blue (lightly myelinated)

**Fig. 5:**
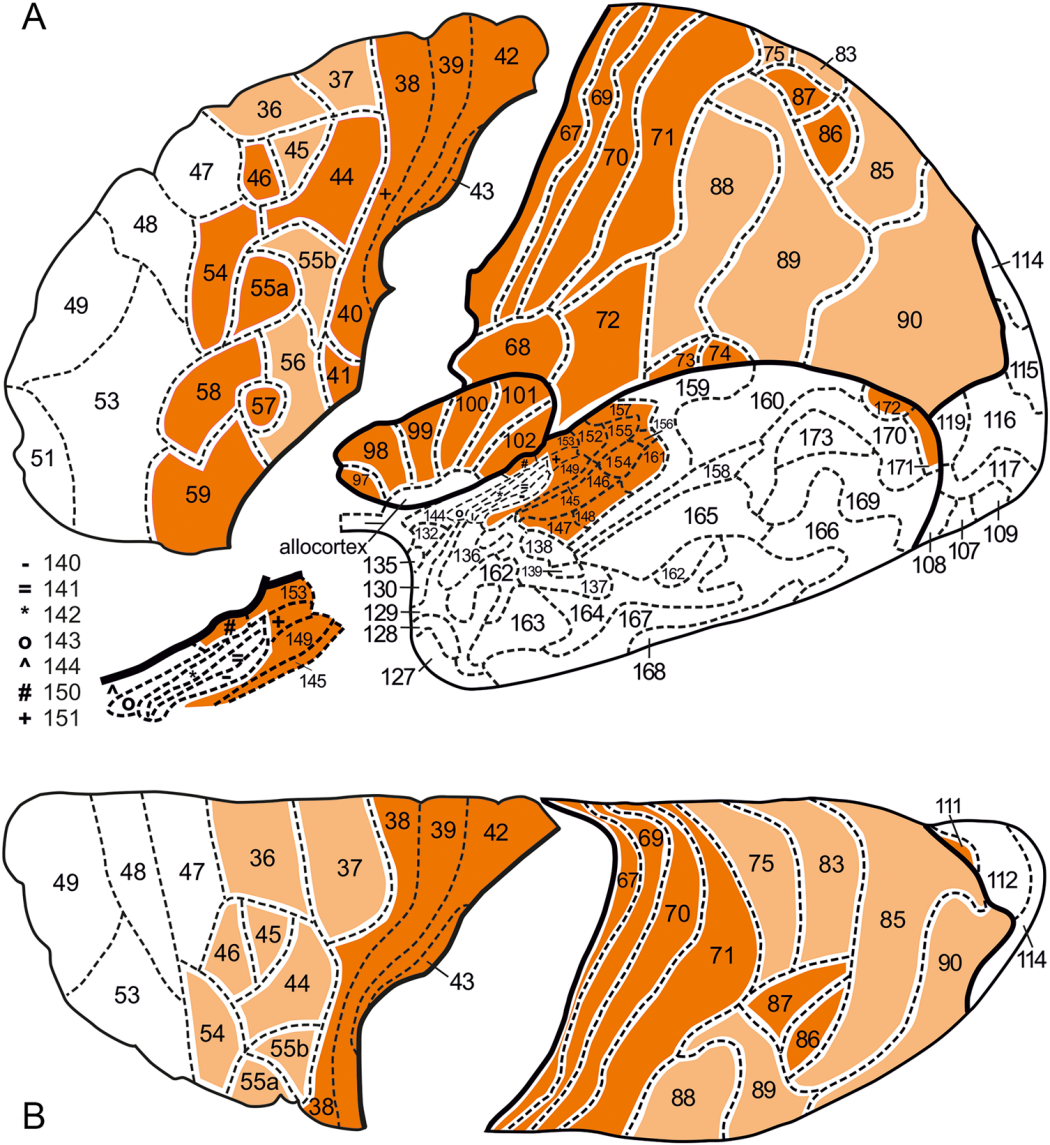
Inflated lateral (**A**) and superior (**B**) views of the 3D Nieuwenhuys and Broere (2023) map. Note that the frontal lobe parts in these two aspects are replaced by 2D maps, illustrating our current insights into the parcellation of these two views. For the significance of the colours in this and the following figures, see text

**Fig. 6:**
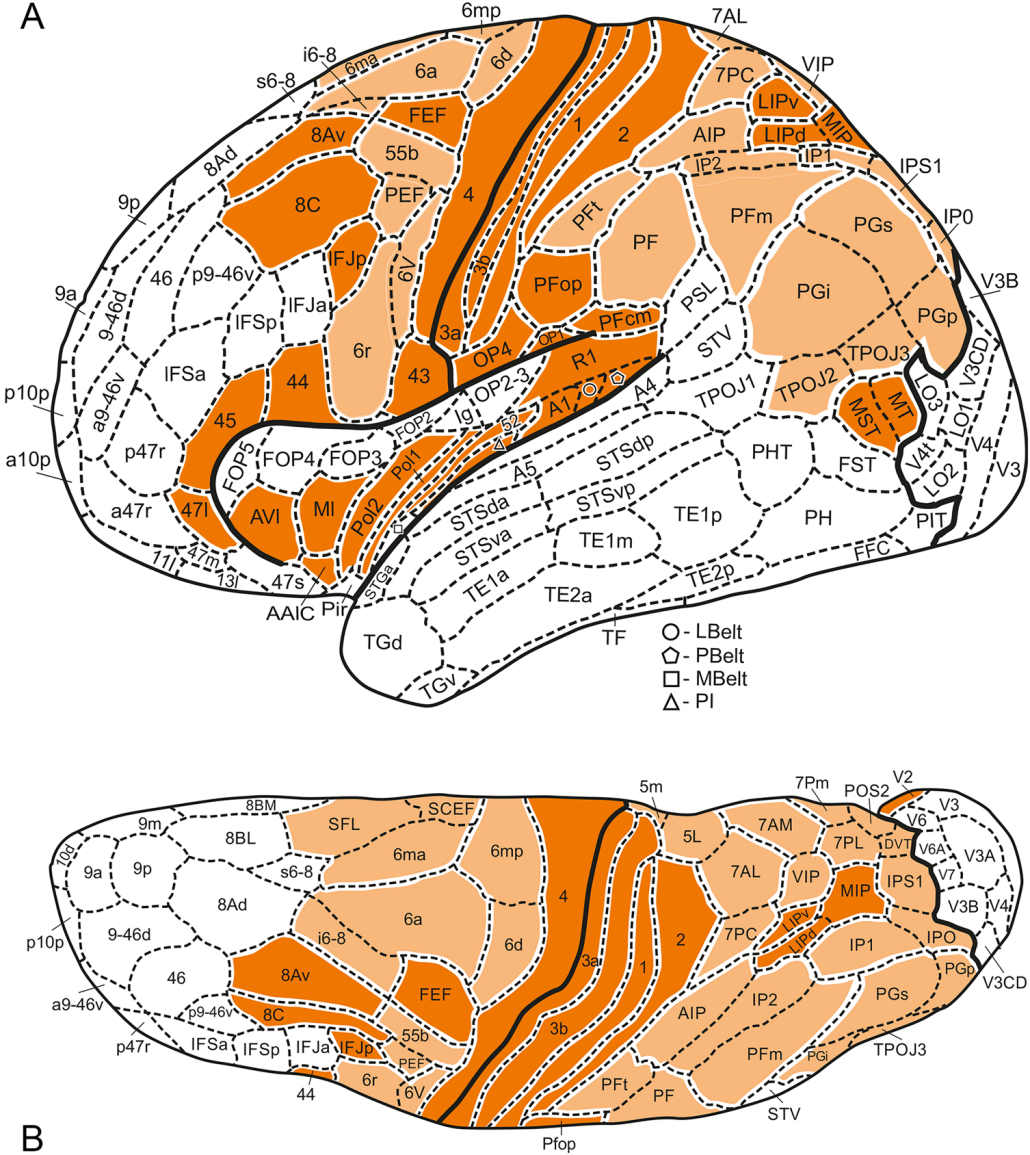
Inflated lateral (**A**) and superior (**B**) views of the 3D Glasser et al. (2016a) map

**Fig. 7:**
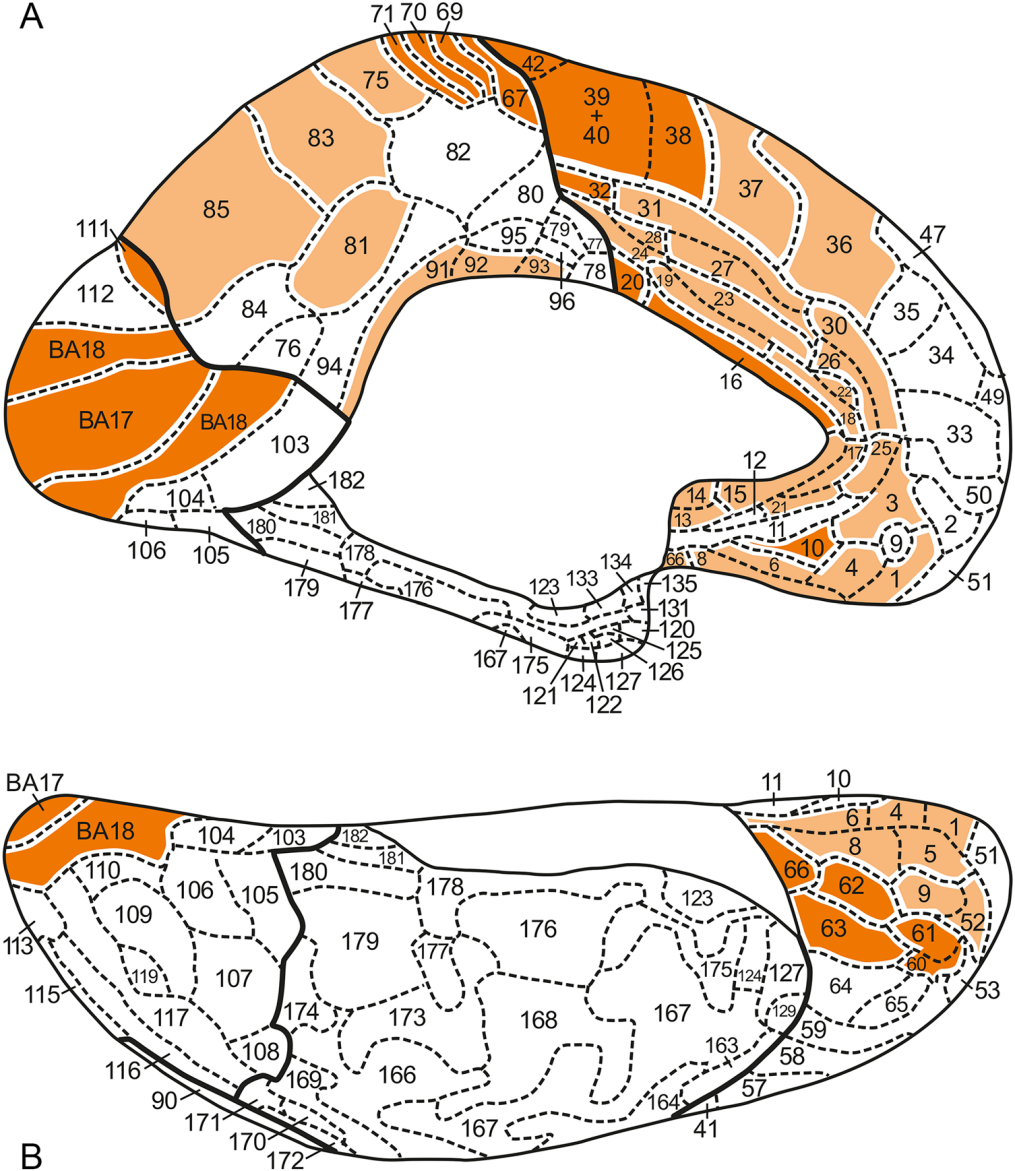
Inflated medial (**A**) and basal (**B**) views of the 3D Nieuwenhuys and Broere (2023) map

**Fig. 8:**
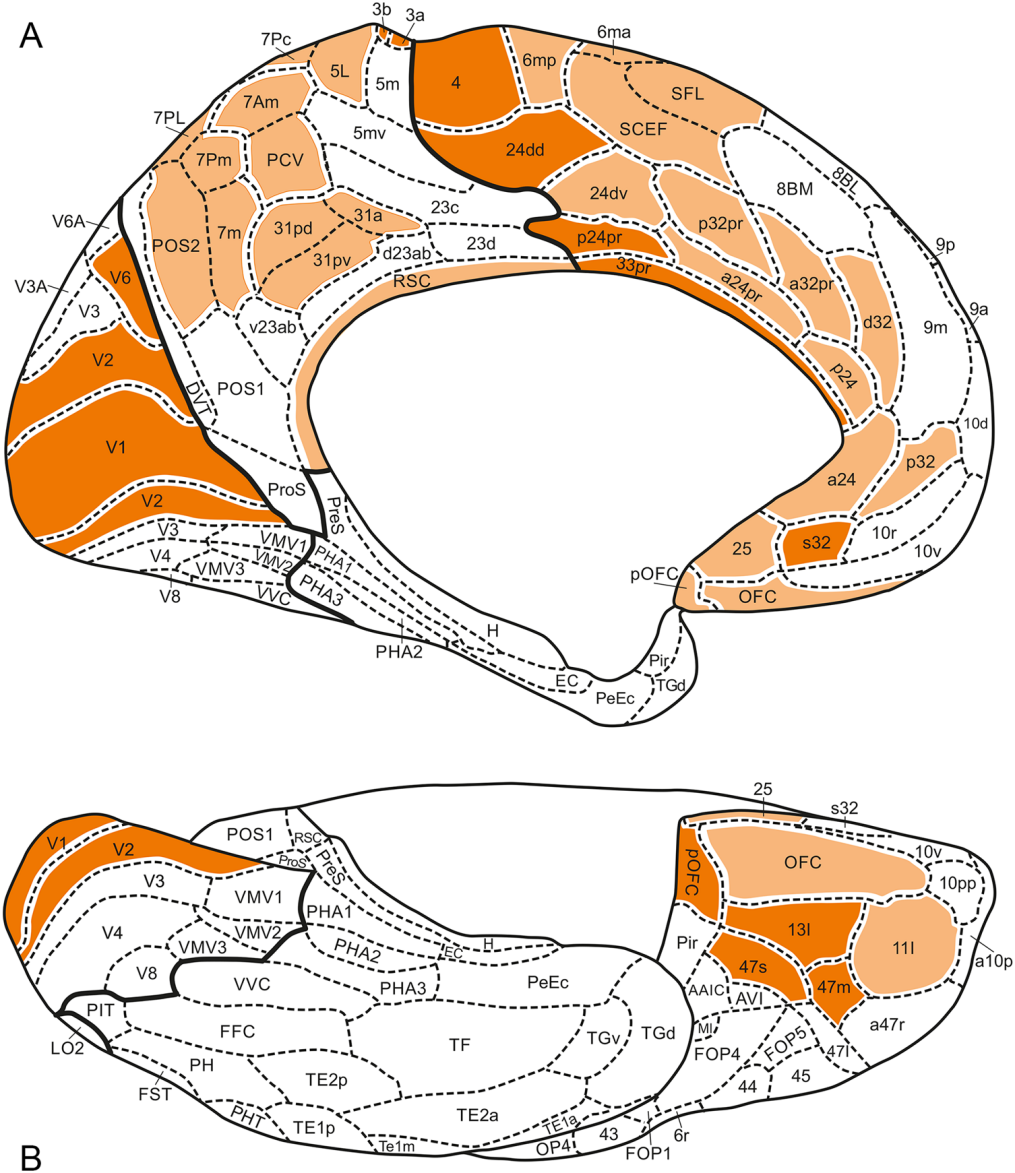
Inflated medial (**A**) and basal (**B**) views of the 3D Glasser et al. (2016a) map

In order to avoid crowding, the cerebral sulci are not included in our maps (Figs. 5, 6, 7, 8). However, they are clearly visible in a special map, showing a group average midthickness surface of 1071 multimodally aligned young adults from the Human Connectome Project (Fig. 2), as well as in our standard atlas of the Colin brain (Nieuwenhuys et al. 2015a; Figs. 2–6), of which here the lateral view of the brain is shown (Fig. 3). The cerebral sulci are also included in our 2D’15 map (Nieuwenhuys et al. 2015a).

As regards the relative myelin content, in 2017, we published a map of the human neocortex, showing the overall myelin content of the individual architectonic areas, based on the studies of Hopf (1954, 1956) and Hopf and Vitztum (1957), prominent members of the Vogt-Vogt school (Nieuwenhuys and Broere 2017). Moreover, computerized renderings of the myelin density in the various areas of the HCP map were available (see the supplementary information, attached to Glasser et al. 2016a).

These maps showed that in the human cortex the primary sensory and motor areas are heavily myelinated, and that the remaining regions of the cortex contain several other densely myelinated loci, each consisting of one or more myeloarchitectonic areas. Figure 4 shows one of these dark loci, situated in the orbitofrontal cortex.

The basis for our comparative analysis consisted of four standard views (lateral, superior, medial and basal) of the left hemisphere of the Nieuwenhuys and Broere (NB), and the Glasser et al. (HCP) maps (Figs. 5, 6, 7, 8).

## Results

During our analysis, we detected the following four types of concordance: (a) a particular NB area corresponds with a single HCP area; (b) a particular NB area corresponds with a set of HCP areas; (c) a particular HCP area corresponds with a set of NB areas, and (d) a set of NB areas corresponds with a set of HCP areas (but further concordances within these two groups are uncertain). The various entities showing concordance are collectively denoted as loci of concordance.

The results of our analysis are presented in Table 1. All loci of concordances found are collectively colored orange in Figs. 5, 6, 7, 8).

**Table 1:**
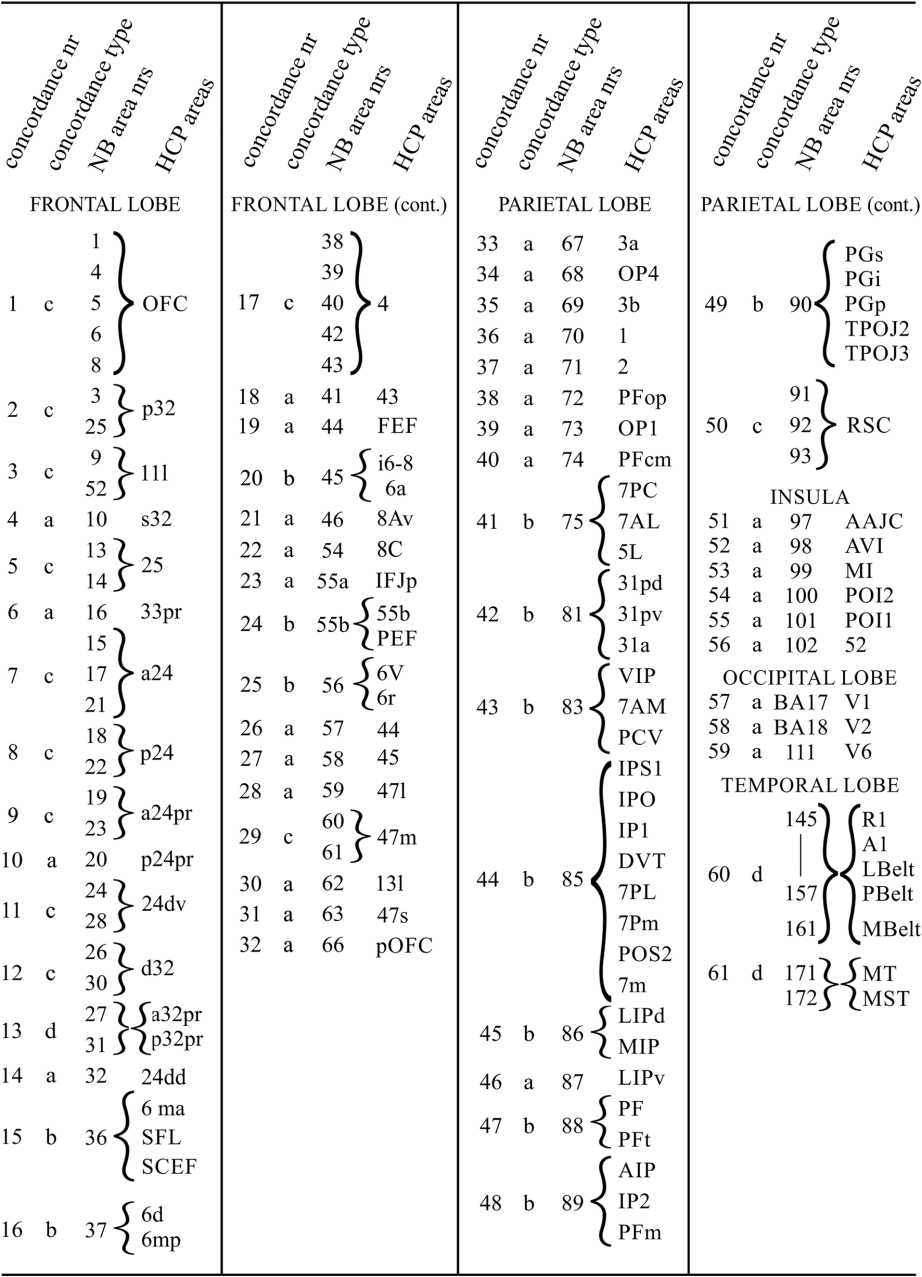
Loci of Concordance in Map NB and Map HCP

### The frontal lobe

As has already been mentioned, we do not trust the correctness of the parcellation of the frontal lobe, as shown in Nieuwenhuys and Broere (2023: Figs. 4A, B) anymore. In these figures, the areas 38–40 and 42–43 are extraordinarily wide, with spatial consequences for the areas located in the remainder of the lobe. A preliminary reanalysis of our material, including the study of Hopf (1956), indicates that the antero–posterior dimensions of the areas mentioned are much smaller, and that area 40 is not only smaller, but also forms part of area 38. Because of these discrepancies, we decided to replace the lateral and superior views of the frontal lobe in our original 3D parcellation of the frontal lobe, by 2D maps, reflecting our new insights (Fig. 5A, B). The NB areas 38 + 40, 39, 42 and 43 in these new maps are all heavily myelinated, and are obviously collectively concordant with the likewise heavily myelinated HCP area 4. A phenomenon, described by Vogt and Vogt (1919) and Hopf (1956), viz*.* that in the frontal lobe the overall density of the myelin decreases stepwise from the precentral (motor) cortex to the frontal pole, could be confirmed by Glasser et al. (2016a, Fig. 10B of the Supplemental Neuroanatomical Results). It may be added that the areas that receive the large sensory projections (somatosensory, auditory, visual) are also heavily myelinated, and that the myelination decreases stepwise from all of these areas.

Several of the areas, observed on the lateral aspect of the frontal lobe, including NB areas 36, 37, 38 + 40, 39 and 42, and their HCP concordances (*cf*. Table 1) extend, via the superior aspect (Figs. 5B, 6B), over the medial aspect of the frontal lobe (Figs. 7A, 8A). The remaining concordances detected on the medial aspect of the frontal lobe are mainly confined to the zone adjacent to the corpus callosum (Figs. 7A, 8A).

It has already been mentioned that the human neocortex contains, apart from the primary sensory and motor areas, several other densely myelinated loci, each consisting of one or more areas. One of these dark loci is found in the orbitofrontal cortex, and consists of NB areas 60 + 61 (Fig. 4A) and HCP area 47m (Fig. 4B). This cluster facilitated the identification of some adjacent areas, viz*.* NB areas 62 and 63 and HCP areas 13l and 47s.

Two loci of concordance occupy the medial part of the orbitofrontal cortex. These loci are represented by the HCP areas OFC and pOFC (Fig. 5B). HCP area OFC (Fig. 8A, B) is concordant with the set of NB areas 1, 4, 5, 6, 8 (Fig. 7A, B), whereas HCP area pOFC (Fig. 8B) is concordant with NB area 66 (Fig. 7B). Note that HCP area OFC is located in a region where the MRI-based parcellation is inaccurate because of MRI signal loss. The olfactory sulcus (Figs. 2, 3) occupies a central position in the concordant NB loci 1 + 4 + 5 + 6 + 8 and HCP locus OFC.

A large number of concordances could be determined in the *parietal lobe.* The postcentral gyrus, bounded anteriorly by the central sulcus and posteriorly by the postcentral sulcus (Figs. 2, 3), contains four elongated, strip-like areas, the NB areas 67, 69, 70 and 71 (Fig. 5A, B), which are evidently concordant with the HCP areas 3a, 3b, 1 and 2, respectively (Fig. 6A, B). The superior parietal lobule, which is partly separated from the inferior parietal lobule by the intraparietal sulcus (Fig. 2), contains a heavily myelinated locus, consisting of the NB areas 86 and 87 (Fig. 5A, B), concordant with the HCP areas LIPd + MIP and LIPv, respectively (Fig. 6A, B). Some of the areas in the inferior parietal lobule show one-to-one concordances in the two maps, as *e.g*. NB area 68 (Fig. 5A) with HCP area OP4 (Fig. 6A), and NB area 72 (Fig. 5A) with HCP area PFop (Fig. 6A), but by far most of the other areas in this lobule, and all remaining areas in the superior parietal lobule, show one-to-two or one-to-many concordances. Here, we confine ourselves to two examples: NB area 83 (Fig. 5A, B) corresponds with HCP areas VIP + 7AM + PCV (Figs. 6A, B), and NB area 90 (Fig. 5A, B) corresponds with HCP areas PGi + PGs + PGp + TPOJ2 + TPOJ3 (Fig. 6A, B). Note, however, that VIP has a substantially higher myelin content than 7AM and PCV in the MRI-based myelin maps. On the medial side of the parietal lobe, the superior parietal lobule and the precuneus are occupied by medial extensions of the NB areas 75, 83 and 85 (Fig. 7A) and their concordant composite HCP loci (Fig. 8A). It is noteworthy that HCP area POS2 is substantially more heavily myelinated than other portions of NB area 85 (Glasser and Van Essen 2011) and this large hotspot of heavier myelination appears to have been missed by the classical myeloarchitectonic maps, perhaps because the parieto-occipital sulcus was the border zone between several of the maps that confined themselves to the parietal or occipital lobes (Vogt 1911; Lungwitz 1937; Batsch 1956) but was itself perhaps not studied intensively. Finally, the pericallosal NB areas 91, 92 and 93 (Fig. 7A), which are more heavily myelinated than their adjacent areas, are collectively concordant with HCP area RSC (Fig. 8A).

In the *insular lobe* six concordances could be detected: NB areas 97, 98, 99, 100, 101 and 102 (Fig. 5A) correspond with HCP areas AAJC, AVI, MI, Pol2, Pol1 and 52, respectively (Fig. 6A).

### The occipital lobe

As is well known, Brodmann (1909) divided the human occipital cortex into three concentrically arranged areas, the area striata (BA17), area occipitalis (BA18) and area preoccipitalis (BA19). Areas BA17 and BA18 are in all classical mapping studies of the human cortex recognized as distinct entities (*cf.* Braak 1980); hence, NB included these areas in their map (Fig. 7A, B). They are obviously concordant with the HCP areas V1 and V2 (Figs. 6B; 8A, B). Area 17BA/V1 is found within the calcarine sulcus (Fig. 2). Glasser et al. (2016a, Fig. 2B of the Supplemental Neuroanatomical Results) found that both of these areas are heavily myelinated, area V1 even stronger than V2. Contrary to areas BA17 and BA18, area BA19 has appeared to be heterogeneous. Lungwitz (1937), who studied its myeloarchitecture, divided it into no less than 17 different areas. These are included in our NB map, where they are numbered 103–119 (Figs. 5A, B and 7A, B) They correspond collectively with the 13 HCP areas V3, V3A, V3B, V4, V4t, V6, V6A, V7, V8, VMV1, VMV2, VMV3 and VVC (Figs. 6A, B and 8A, B). We failed, however, to detect concordances between the individual areas of these two parcellations, except for NB area 111 (Fig. 7A), which corresponds topologically with HCP area V6 (Fig. 8A).

### The temporal lobe

The parcellation of the temporal lobe of the NB map (Figs. 5A and 7A, B), appeared to be so different from that of the HCP map (Figs. 6A and 8A, B), that we succeeded in finding only two loci of concordance in these two maps, one in the posterolateral temporal region, the other in the planum temporale. The posterolateral locus is composed of the NB areas 171 + 172 (Fig. 5A), and of the HCP areas MT + MST (Fig. 6A). The areas forming this locus are characterized by a very high myelin content. The likewise very heavily myelinated locus in the planum temporale, is formed by the primary auditory cortex, which includes in the NB map no less than 14 areas: 145–157 + 161 (Fig. 5A). This cortex is in the HCP map represented by 5 areas: R1, A1, LBelt, PBelt and MBelt (Fig. 6A).

In total, we detected 61 loci of concordance, 33 of type a, 13 of type b, 12 of type c, and 3 of type d. All concordances of type a (*i.e.* one-to-one), and all concordances, primarily based on heavy myelination, are presented in Figs. 5, 6, 7, 8 in dark orange; all remaining concordances in light orange.

## Discussion

The construction of a probabilistic, unified, canonical parcellation of the human cerebral cortex, to be used as a gold standard or ground truth in all morphological and functional studies on that organ, would be a major scientific achievement. In this paper, we present a detailed comparison of two recent 3D maps of the human cortex, both of which comprising 180 areas, as a first step towards that goal. We consider all of the concordant loci, indicated in dark orange in Figs. 5, 6, 7, 8, as serious candidates for including in the unified map. The remaining concordant loci are also candidates, but require further analysis.

## Limitations

This study represents the authors’ best efforts using classical methods and the criteria discussed above at making a detailed consensus comparison between the NB composite map of the Vogt-Vogt myeloarchitectonic studies and the HCP multimodal parcellation, spanning over a century of neuroanatomical map making of the human cerebral cortex. That said, there are several important limitations to this study. 1) Although underappreciated in the classical period, it has become clear that human brains are highly variable across individuals, both in their folding patterns and the locations of areas relative to folds. Thus, many of the locations without concordances could simply be due to idiosyncrasies of the individuals studied in the classic maps (whereas the HCP map of cortical areas represents the typical pattern found in 210 precisely aligned individuals. 2) There is increasing awareness of biases in cortical areal maps identified on 2D histological slices due to plane of section effects, something that 3D MRI scans are unaffected by (Fischl and Sereno 2018), but which could affect the concordances reported here. 3) It is now routine to use computational tools to accurately and directly map findings from 3D MRI scans or 2D histology slices onto cortical surface meshes to produce inflated views of the cortical surface, showing the cortex within the depths of the sulci. Although Nieuwenhuys and Broere have done their utmost to bring the classic maps of the Vogt-Vogt school into the modern era, there are many steps in this process that required visual-perceptual judgements both on the parts of the original authors (*e.g.*, regionally expanding cortex to show findings buried in the sulci on 2D surface drawings in their publications) and their later translators that could introduce errors and make concordances more challenging to find. Such errors also influence some alternative maps generated from this material, including Foit et al. 2022 and Mai and Majtanik 2023, and here we focused on the comparison with the NB map, which we were most familiar with. The gold standard approach for comparisons such as those presented here require mapping both datasets (i.e., boundaries from myelin stains and multi-modal MRI) to surface meshes of the individuals being studied and aligning those meshes with surface-based registrations, ideally with features that are closely tied to cortical areas such as myelination or functional MRI. In less variable brain regions, precise comparisons can be made with folding-based surface registration as in Glasser and Van Essen 2011 and Glasser et al. 2016a. Unfortunately, such an approach from the original Vogt-Vogt school source material is not possible; however, MRI technology may continue to advance and enable laminar myeloarchitectonic mapping in living human brains, above and beyond the extant overall cortical myelin content maps, to be compared with the other modalities, such as cortical thickness and resting state and task-based functional MRI. Such advances would make revisiting of the photographs and textual descriptions of the areas defined by the classical maps, guided by the concordances described above, helpful.

## Perspective

The authors consider a detailed comparison of the multimodal map of the human cortex of Glasser et al. (2016a), with the cytoarchitectonic and receptorarchitectonic map of Amunts et al. (2020), as a logical second step in the endeavor here initiated. Such an effort would be dramatically aided by mapping the remaining areas of the Zilles and Amunts school onto individual surface meshes and registering them to the HCP standard mesh to enable the precise comparisons described above, as was previously done for the few areas presented in Fischl et al. 2008, which have already been directly compared to the HCP atlas of areas in Glasser et al. 2016a (see Supplementary Neuroanatomical Results therein). It may be expected that such a comparison will confirm many of the concordances found in the present paper, and will yield numerous new ones.

## Data Availability

This study analyzed already publicly available datasets and generated no new datasets.

## Abbreviations

acg: Anterior cingulate gyrus
ang: Angular gyrus
aog: Anterior orbital gyrus
aos: Arcuate orbital sulcus
calcs: Calcarine sulcus
ces: Central sulcus
cols: Collateral sulcus
cs: Cingulate sulcus
csab: Cingulate sulcus, ascending branch
fg: Fasciolar gyrus
ifgop: Inferior frontal gyrus, opercular part
ifgor: Inferior frontal gyrus, rostral part
ifgt: Inferior frontal gyrus, triangular part
ifs: Inferior frontal sulcus
ipl: Inferior parietal lobule
ips: Intraparietal sulcus
itg: Inferior temporal gyrus
its: Inferior temporal sulcus
los: Lateral orbital sulcus
lotg: Lateral occipitotemporal (fusiform) gyrus
ls: Lateral sulcus
lsab: Lateral sulcus, ascending branch
lsan: Lateral sulcus, anterior branch
mfg: Middle frontal gyrus
mog: Medial orbital gyrus
motg: Medial occipitotemporal (lingual) gyrus
mtg: Middle temporal gyrus
og: Occipital gyri
olfs: Olfactory sulcus
ots: Occipitotemporal sulcus
pcl: Paracentral lobule
pcun: Precuneus
phg: Parahippocampal gyrus
pocg: Postcentral gyrus
pocs: Postcentral sulcus
pog: Posterior orbital gyrus
pos: Parieto-occipital sulcus
prcg: Precentral gyrus
prcs: Precentral sulcus
pron: Preoccipital notch
rhs: Rhinal sulcus
sfg: Superior frontal gyrus
sfs: Superior frontal sulcus
smg: Supramarginal gyrus
spl: Superior parietal lobule
stg: Superior temporal gyrus
strg: Straight gyrus
sts: Superior temporal sulcus
stsp: Superior temporal sulcus, posterior part
